# Understanding the Objective Structured Clinical Examination (OSCE) Through the Lens of Medical College Interns: A Knowledge, Attitude, and Practice (KAP)-Based Study in a Private Medical College in Central India

**DOI:** 10.7759/cureus.105837

**Published:** 2026-03-25

**Authors:** Anjali Chhari, Swati Sharma, Juhi Loya

**Affiliations:** 1 Obstetrics and Gynaecology, Mahaveer Institute of Medical Sciences and Research, Bhopal, IND

**Keywords:** assessment, attitude, knowledge, objective structured clinical exam (osce), osce

## Abstract

Introduction: The Objective Structured Clinical Examination (OSCE) is a widely accepted method for assessing clinical competence in medical education. The OSCE is a standardized, objective method to evaluate the clinical skills of medical students, encompassing history-taking, examination skills, and communication. As medical education shifts towards competency-based curricula, understanding student perspectives on OSCE becomes vital. Knowledge, attitude, and practice (KAP) surveys provide valuable insight into how students perceive and prepare for this form of assessment. This study aimed to assess the KAP of interns regarding OSCE.

A structured KAP questionnaire was administered to 150 interns from a tertiary medical college. Data were analysed using descriptive statistics. Results indicated that while the majority of students had adequate knowledge and a positive attitude toward OSCE, practice preparedness varied. The findings highlight the need for enhanced orientation and training to improve practical readiness for OSCE.

Materials and methods: A cross-sectional study was conducted among 150 interns at Mahaveer Institute of Medical Sciences and Research, Bhopal, India. Responses were scored and analysed using SPSS Statistics for Windows, Version 25.0 (Released 2017; IBM Corp., Armonk, NY, USA). Descriptive statistics were used to summarize the survey data.

Results: Out of 150 students, 138 completed the questionnaire (response rate: 92%). For knowledge, 78% demonstrated good knowledge of the OSCE components and structure. For attitude, 85% expressed a positive attitude, believing the OSCE to be a fair and useful assessment method. For practice, only 60% reported engaging in regular practice or mock OSCEs. Common barriers included limited access to practice materials and time constraints.

Conclusion: Interns possess good knowledge and a positive attitude towards OSCE but require more opportunities for hands-on practice. Medical colleges should focus on structured OSCE training programs to improve students' clinical competency and assessment readiness.

## Introduction

Assessment of medical students plays a pivotal role in shaping medical education. George E. Miller emphasized that changes in examination systems often influence learning more profoundly than curricular reforms alone. This clearly demonstrates the importance of assessment. Traditional assessment methods relied predominantly on long-case examinations, viva voce, and essay-based written tests. Although these methods assessed theoretical knowledge, they were often subjective, lacked standardization, were examiner-dependent, and were limited in evaluating clinical competence and communication skills [1].

With the evolution of medical education and the emergence of formative assessment strategies, medical education has gradually shifted toward evaluating clinical skills, professionalism, communication, and competency in a structured and unbiased manner. This transformation aligns with the philosophy of competency-based medical education (CBME), which emphasizes measurable learning outcomes, continuous feedback, and development of professional competence [2].

In this context, the Objective Structured Clinical Examination (OSCE), introduced by Ronald Harden in 1975, has emerged as a reliable and standardized method for assessing clinical competence. The OSCE uses multiple structured stations with objective marking schemes to evaluate clinical reasoning, psychomotor skills, and communication abilities while minimizing examiner bias [3].

The OSCE has now become an integral component of medical curricula worldwide because of its ability to simulate real-life clinical scenarios and provide fair and reliable assessment of practical skills [4]. The implementation of CBME guidelines by the National Medical Commission (formerly Medical Council of India) in India formally incorporated the OSCE as an assessment modality in undergraduate medical education, reinforcing its role in developing competent Indian Medical Graduates [2].

Understanding the knowledge, attitudes, and practices (KAP) of interns who have taken the OSCE toward OSCE is essential for enhancing the effectiveness of this examination in medical education. A positive attitude and adequate preparation not only influence the performance of students but also reflect their confidence and readiness to enter clinical practice. Therefore, assessing these KAP dimensions allows educators to identify potential gaps in knowledge and attitudes that may affect the performance of students.

## Materials and methods

The study was conducted at Mahaveer Institute of Medical Sciences and Research, Bhopal, India from January 10 to January 20, 2026 and received approval from the external Institutional Review Board (approval number PCMS/OD/PS/IEC/2026/264).

This study employed a cross-sectional KAP design to collect data from interns of the Medical College, targeting a total of 150 interns (who have appeared in the OSCE in the final year part 2 examination). The study utilized a self-administered questionnaire that was delivered by Google Forms (Appendix). Items of the questionnaire were constructed after reviewing prior KAP-based OSCE studies and were validated through pilot testing with a small group of interns for clarity and reliability before final administration. The students completed the questionnaire anonymously with prior consent and submitted them to the investigators within one week.

A validated, self-administered KAP questionnaire consisted of demographic details, 14 knowledge questions, 15 attitude statements, and 15 practice-related items. The questionnaire consisted of closed-ended multiple-choice questions that were formulated by experts in the field. The questionnaire items were adapted from previously published OSCE perception and KAP studies in medical education [5]. The questionnaire was pilot-tested for clarity and reliability before administration. Responses were scored and analysed using SPSS Statistics for Windows, Version 25.0 (Released 2017; IBM Corp., Armonk, NY, USA). Descriptive statistics were applied to summarize the data, and correlation analyses were used to explore potential relationships between variables, such as the correlation between knowledge and attitudes towards the OSCE.

This study aimed to assess the attitudes towards this examination format and reported practices in preparation for the OSCE among interns at a medical school in India. The findings of this study provide valuable insight into the KAP of interns towards the OSCE, which can inform the development of targeted interventions to enhance the effectiveness of this assessment tool in medical education in India.

## Results

Demographic profile of respondents

A total of 138 interns out of 150 interns consented to participate in the study. Among the participants, 73 (52.9%) were male and 65 (47.1%) were female.

Overall KAP scores

The mean knowledge score among interns was 1.68 ± 0.36, indicating low to moderate knowledge levels with minimal variability. The attitude score demonstrated the highest mean value at 3.84 ± 1.11, reflecting generally positive perceptions toward the OSCE, though with considerable inter-individual variation. The practice score was 1.75 ± 0.66, comparable to the knowledge score but with greater dispersion, suggesting inconsistency in practical preparedness (Table 1).

**Table 1:** Overall Knowledge, Attitude, and Practice (KAP) Scores

	Knowledge Score	Attitude Score	Practice Score
Mean	1.68012	3.83851	1.74638
Standard Deviation	0.36198	1.10563	0.66256

**Figure 1:**
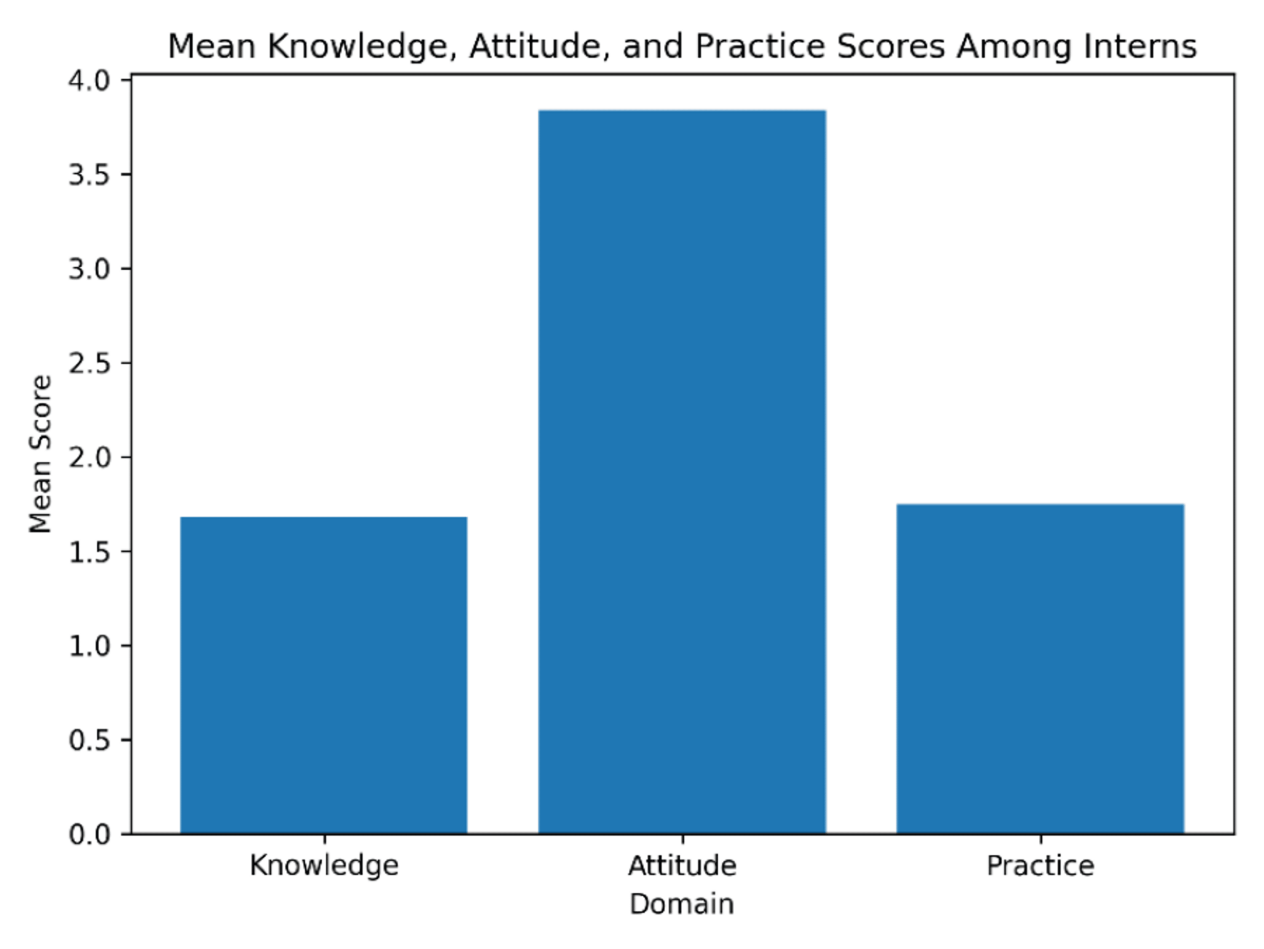
Distribution of Knowledge Scores Among Interns

As illustrated in Figure 1, attitude scores were notably higher than both knowledge and practice scores. This visual trend highlights a discernible gap between positive perception of the OSCE and actual engagement in OSCE-related practice activities.

Participants show the highest average scores in attitude, indicating positive perceptions or intentions. However, both knowledge and practice scores are lower and closer in value, with limited variability in knowledge and slightly more variation in practice. This may suggest a gap between attitude and actual knowledge/practice, highlighting areas for targeted interventions or education.

Descriptive statistics of KAP

Distribution of KAP Scores

Knowledge domain: Knowledge scores were moderately high and tightly clustered, indicating uniform theoretical understanding among interns. Attitude scores were higher on average with a right-skewed distribution, reflecting generally favourable perceptions toward the OSCE. In contrast, practice scores showed a broader spread with a lower mean, indicating inconsistent hands-on preparedness as depicted in Table 2.

**Table 2:** Knowledge Domain Responses Related to the Objective Structured Clinical Examination (OSCE)

Knowledge Item	Correct Response (%)
Full form of OSCE	85.5
Components of OSCE	79
Awareness of scoring system	68.8
Number of examiners	81.2

Attitude domain: Interns exhibited predominantly positive attitudes toward the OSCE. A large proportion perceived the OSCE as objective (88.4%), accurately reflective of clinical skills (87.7%), and a fair assessment method (79.7%). Additionally, 84.0% believed that the OSCE prepares them for real-life clinical scenarios. However, 19.6% of interns perceived the OSCE as stressful (Table 3).

**Table 3:** Attitude Toward the Objective Structured Clinical Examination (OSCE) Among Interns

Attitude Statement	Positive Response (%)
OSCE is objective	88.4
Accurately reflects clinical skills	87.7
Fair assessment method	79.7
Prepares for real-life scenarios	84
OSCE is stressful	19.6

Practice domain: With regard to OSCE-related practices, 87.1% of interns reported actively seeking faculty feedback, while 80.4% engaged in peer practice. Participation in mock OSCEs (79.0%) and emergency scenario practice (79.7%) was high. In contrast, simulation laboratory usage (66.7%) was relatively lower. Participation in OSCE-focused study groups was reported by 71.0% of interns (Table 4).

**Table 4:** Practice Patterns Related to Objective Structured Clinical Examination (OSCE) Preparation

Practice Activity	Participation (%)
Peer practice	80.4
Seeking faculty feedback	87.1
Participation in mock OSCE	79
Use of simulation laboratory	66.7
Emergency scenario practice	79.7
OSCE-focused study groups	71

Distribution of KAP scores

**Figure 2:**
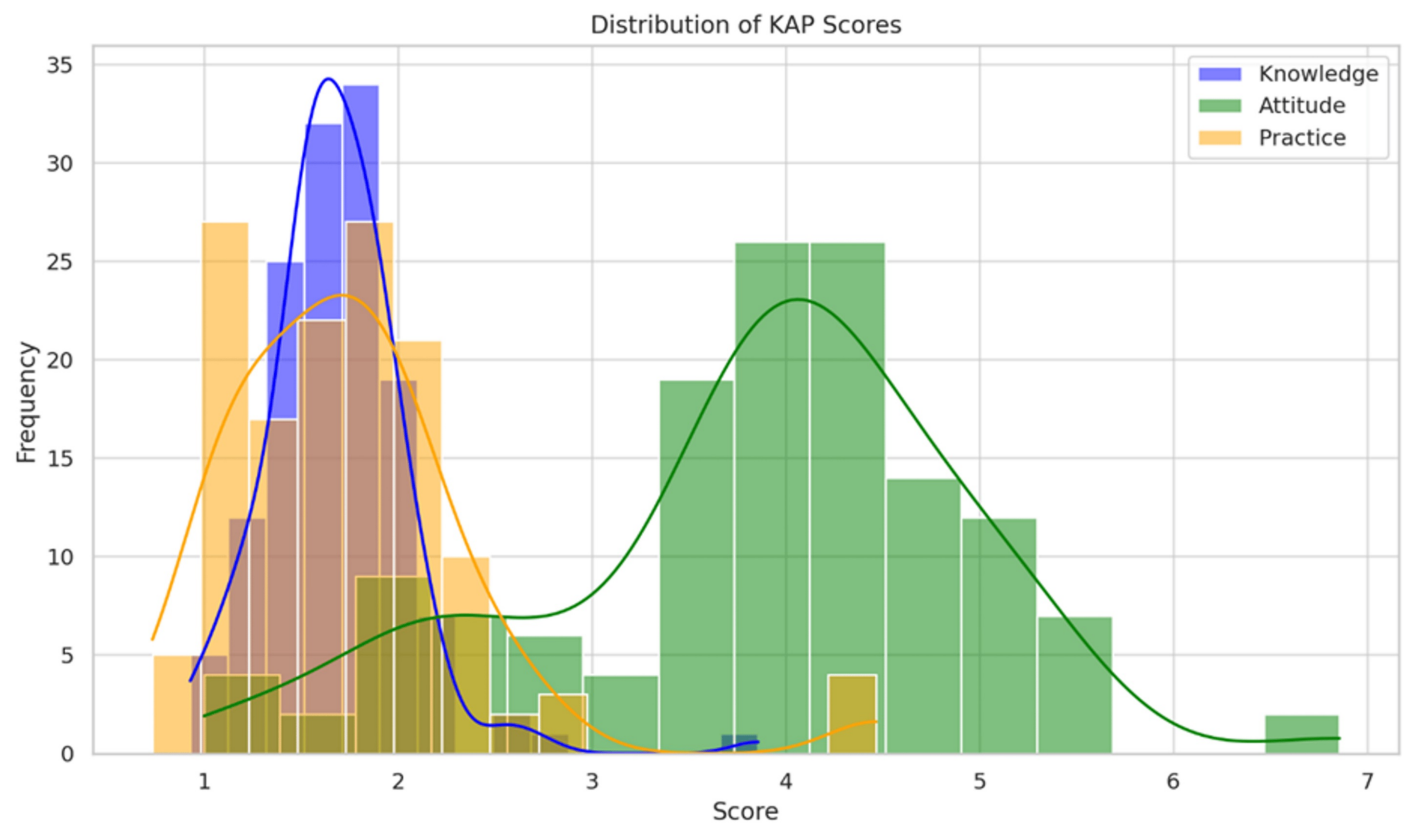
Distribution of Knowledge, Attitude, Practice (KAP) Scores

Knowledge scores were moderately high and tightly clustered, indicating uniform theoretical understanding among interns. Attitude scores were higher on average with a right-skewed distribution, reflecting generally favourable perceptions toward the OSCE. In contrast, practice scores showed a broader spread with a lower mean, indicating inconsistent hands-on preparedness as clearly depicted in Figure 2.

Correlation analysis

Correlation analysis revealed a weak positive correlation between knowledge and attitude scores (r = 0.16, p = 0.056), which did not reach statistical significance. A moderate and statistically significant positive correlation was observed between knowledge and practice scores (r = 0.26, p = 0.002), indicating that interns with higher knowledge levels were more likely to engage in OSCE-related practice activities. The correlation between attitude and practice scores was very weak and non-significant (r = 0.10, p = 0.237) (Table 5).

**Table 5:** Correlation Between Knowledge, Attitude, and Practice Scores p value - probability value.

Variables	Correlation Coefficient (r)	p-value
Knowledge vs Attitude	0.16	0.056
Knowledge vs Practice	0.26	0.002*
Attitude vs Practice	0.1	0.237

**Figure 3:**
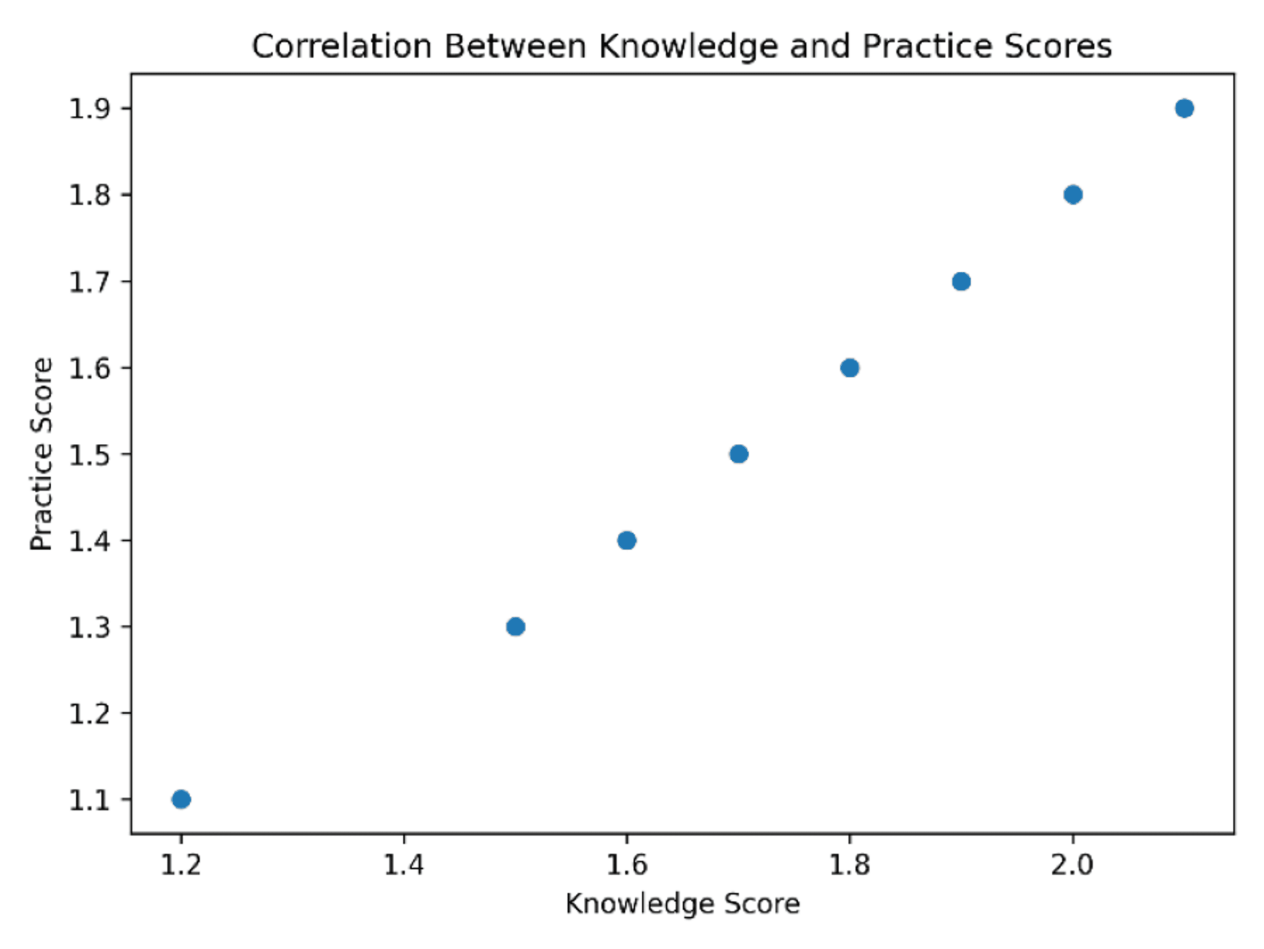
Correlation Between Knowledge and Practice Scores

The relationship between knowledge and practice scores is visually demonstrated in Figure 3, which illustrates an upward trend, supporting the statistical findings.

The study indicates moderately high knowledge and positive attitudes towards the OSCE among interns. Despite adequate familiarity, students reported notable stress levels. Previous OSCE exposure significantly improved practice scores. Incorporating mock OSCEs, frequent feedback, and improved faculty support may enhance OSCE preparedness.

**Figure 4:**
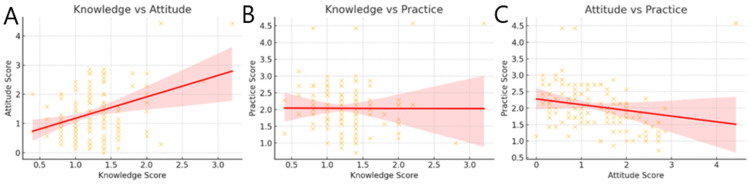
Correlation Between Knowledge and Attitude, Knowledge and Practice, and Attitude and Practice Scores

Correlation analysis demonstrated a weak positive relationship between knowledge and attitude scores (r = 0.16), indicating that higher knowledge levels were only slightly associated with more favorable attitudes toward the OSCE (Figure 4A). A moderate positive correlation was observed between knowledge and practice scores (r = 0.26), suggesting that students with better knowledge were more likely to engage in OSCE-related practice activities (Figure 4B). In contrast, the relationship between attitude and practice scores was very weak (r = 0.10), implying that positive perceptions alone did not necessarily translate into improved practical preparation, interpretating that students with better knowledge tend to practice more (0.26), though not strongly and attitude has the weakest direct link with practice (0.1), suggesting perception alone may not lead to action (Figure 4C).

Gap analysis

A subgroup analysis identified 31 interns who demonstrated high knowledge scores but low practice scores, highlighting a significant knowledge-practice gap. Despite adequate conceptual understanding of the OSCE, these interns lacked sufficient hands-on engagement, suggesting potential barriers such as limited structured practice opportunities, restricted access to simulation facilities, or time constraints during internship.

## Discussion

The findings of this study align with existing literature demonstrating generally positive perceptions of the OSCE among medical students and interns. Previous studies show that participants perceived OSCE positively due to its unique features related to structure, logical sequence, standardized scoring tools, and coverage of a broad spectrum of critical clinical skills [6]. Implementing mock OSCE sessions and simulation-based learning environments may bridge the gap between theoretical knowledge and practical performance. Similar studies conducted in Ethiopia and other countries have reported comparable relationships between knowledge and preparation practices toward the OSCE [7].

Students in this study demonstrated adequate knowledge and favourable attitudes toward the OSCE; however, practical preparedness remained relatively lower. Earlier studies have reported that lack of structured orientation, insufficient exposure to mock examinations, and limited simulation-based learning opportunities may contribute to this gap [8]. Incorporating structured OSCE training sessions, peer-learning opportunities, and formative feedback mechanisms can improve students’ confidence and competence [9].

Several factors may have contributed to the observed results. The positive attitudes most students have towards the OSCE might be a reflection of the perceived benefits of such assessments in improving clinical skills, as previously noted in research focusing on nursing students' experiences. Contrarily, the inadequacy in preparation could be due to a lack of tailored orientation programs or insufficient exposure to real-life clinical scenarios that mimic OSCE conditions. The results indicate a strong theoretical understanding and favourable perception of the OSCE among students. However, practical preparedness remains suboptimal.

This study has a few limitations. First, it relied on self-reported responses, which may be affected by recall bias and social desirability bias. Second, the study was conducted at a single institution, limiting generalizability to other medical colleges with different teaching methods or assessment formats. Third, the questionnaire assessed perceived practices rather than objectively measured performance in OSCE stations. Finally, the cross-sectional design prevents establishing causal relationships between knowledge, attitudes, and practice behaviours. Future multi-centre longitudinal studies with objective performance assessment are recommended to validate these findings.

## Conclusions

In summary, this KAP study highlights the good level of knowledge among Interns regarding the OSCE, along with their largely positive attitudes. However, the results indicate a pressing need for enhanced preparedness initiatives in the future. Understanding the KAP of interns toward the OSCE is crucial for improving the efficacy of examinations and ensuring students transition successfully into competent medical practitioners. The qualitative aspects of KAP and the impact of specific interventions on OSCE performance should be explored in the future. 

An educational environment that emphasizes effective learning strategies, bedside learning, and hands-on clinical exposure can significantly improve student performance in the OSCE, thereby strengthening the future quality of medical education and patient care. Additionally, ongoing evaluation and refinement of existing preparatory programs are essential to address the dynamic nature of clinical assessments and the evolving healthcare demands.

## Appendices

INTERNS

  Please take a few minutes to complete the following questionnaire. Your responses will help us better understand the knowledge, attitudes, and practices of Internds regarding the Objective Structured Clinical Examination (OSCE).

 For each question, please tick the most appropriate answer or provide a brief response where required. Your participation is voluntary, and all responses will be kept confidential. Thank you for your time and cooperation.

SECTION A: DEMOGRAPHIC INFORMATION

1.      Name                           :                                                                   

2.      Age                              :                                                                   

3.      Gender                        :                                                                   

4.      Institute                       :                                                                   

5.      Year of Study              :                                                                   

6.      Have you participated in an OSCE before?

o    Yes

o    No

SECTION B: KNOWLEDGE

1.      What does OSCE stand for?

o    Objective Structured Clinical Examination

o    Objective Standardized Clinical Examination

o    Observational Structured Clinical Evaluation

o    Other (please specify):                       

2.      How familiar are you with the OSCE format?

o    Very familiar

o    Somewhat familiar

o    Not familiar at all

3.      What are the key components assessed in OSCE?

o    Clinical skills

o    Communication skills

o    Procedural skills

o    Theoretical knowledge

o    Ethical decision-making

o    Time management

4.      How is performance typically evaluated in OSCE?

o    Through standardized checklists

o    Through examiner’s subjective judgment

o    By peer review

o    I don’t know

5.      What types of stations are commonly included in OSCE?

o    History taking

o    Physical examination

o    Counseling

o    Procedural skills

o    Diagnostic interpretation

o    Emergency management

6.      How much time is usually allotted per station in an OSCE?

o    5 minutes

o    8 minutes

o    10 minutes

o    15 minutes

o    I don’t know

7.      Is OSCE a summative or formative assessment?

o    Summative

o    Formative

o    Both

o    I don’t know

8.      Are you aware of the scoring criteria used in OSCE?

o    Yes

o    No

9.      How many examiners typically assess a student in each OSCE station?

o    One

o    Two

o    More than two

o    I don’t know

10.  What is the main objective of OSCE?

o    To assess theoretical knowledge

o    To evaluate clinical and communication skills

o    To test procedural competency

o    To assess ethical reasoning

o    I don’t know

11.  Are you aware that feedback is often provided after OSCEs?

o    Yes

o    No

12.  Have you been informed about the competencies that OSCEs assess?

o    Yes

o    No

13.  Do you think OSCE is more objective than traditional clinical examinations?

o    Yes

o    No

o    Not sure

14.  What resources have you used to learn about OSCE?

o    Lectures

o    Workshops

o    Textbooks

o    Online courses/tutorials

o    Peer discussions

o    Mock exams

SECTION C: ATTITUDE

1.      How do you perceive OSCE as a method of assessment?

o    Very effective

o    Effective

o    Neutral

o    Ineffective

o    Very ineffective

2.      Do you believe OSCE accurately reflects a student’s clinical skills?

o    Strongly agree

o    Agree

o    Neutral

o    Disagree

o    Strongly disagree

3.      How stressful do you find OSCE compared to other examinations?

o    Much more stressful

o    More stressful

o    Equally stressful

o    Less stressful

o    Not stressful

4.      Do you think OSCE is a fair assessment method?

o    Strongly agree

o    Agree

o    Neutral

o    Disagree

o    Strongly disagree

5.      How confident do you feel about performing well in OSCE?

o    Very confident

o    Confident

o    Neutral

o    Not very confident

o    Not confident at all

6.      Would you prefer OSCE over traditional clinical exams?

o    Yes

o    No

o    Not sure

7.      Do you believe OSCE should be a mandatory part of medical training?

o    Strongly agree

o    Agree

o    Neutral

o    Disagree

o    Strongly disagree

8.      How well do you think OSCE prepares you for real-life clinical situations?

o    Very well

o    Well

o    Neutral

o    Poorly

o    Very poorly

9.      Do you feel the feedback received after OSCE is helpful for improvement?

o    Strongly agree

o    Agree

o    Neutral

o    Disagree

o    Strongly disagree

10.  How do you perceive the role of OSCE in your overall medical education?

o    Essential

o    Important

o    Neutral

o    Not very important

o    Unnecessary

11.  Do you think OSCEs help identify areas of clinical weakness?

o    Yes

o    No

o    Not sure

12.  Would you recommend that OSCE be implemented in other medical disciplines?

o    Yes

o    No

o    Not sure

13.  How do you feel about the level of difficulty in OSCE stations?

o    Too difficult

o    Difficult

o    Moderate

o    Easy

o    Too easy

14.  Do you believe OSCE efficiently uses time for both students and faculty?

o    Strongly agree

o    Agree

o    Neutral

o    Disagree

o    Strongly disagree

15.  What changes would you suggest to improve the OSCE process?

o                                                                                                                                                                                          

SECTION D: PRACTICE

1.      How often do you practice clinical skills that are tested in OSCE?

o    Daily

o    Weekly

o    Monthly

o    Rarely

o    Never

2.      What methods do you use to prepare for OSCE?

o    Self-study

o    Group study

o    Simulation practice

o    Attending workshops

o    Mock OSCE exams

o    Faculty guidance

3.      How many hours per week do you dedicate to OSCE preparation?

o    Less than 1 hour

o    1-3 hours

o    4-6 hours

o    7-10 hours

o    More than 10 hours

4.      Do you participate in peer-led OSCE practice sessions?

o    Always

o    Often

o    Sometimes

o    Rarely

o    Never

5.      How often do you seek feedback from faculty or peers on your OSCE performance?

o    Always

o    Often

o    Sometimes

o    Rarely

o    Never

6.      How effective do you find mock OSCE exams in your preparation?

o    Very effective

o    Effective

o    Neutral

o    Ineffective

o    Very ineffective

7.      How frequently do you use simulation labs for OSCE preparation?

o    Frequently

o    Occasionally

o    Rarely

o    Never

o    We don’t have simulation labs

8.      Do you review OSCE stations and cases after exams to improve?

o    Always

o    Often

o    Sometimes

o    Rarely

o    Never

9.      Have you created or participated in OSCE study groups?

o    Yes

o    No

10.  How do you prioritize your study for OSCE stations?

o    Based on previous experience

o    Faculty recommendations

o    Commonly tested stations

o    Random selection

o    Other (please specify):                       

11.  Do you keep a logbook or notes specifically for OSCE preparation?

o    Yes

o    No

12.  How do you approach time management during an OSCE station?

o    Practice timing each task

o    Follow a set routine

o    Rely on intuition

o    Struggle with time management

o    Other (please specify):                       

13.  Do you practice the communication skills required for OSCE?

o    Always

o    Often

o    Sometimes

o    Rarely

o    Never

14.  How often do you simulate emergency scenarios as part of your OSCE preparation?

o    Always

o    Often

o    Sometimes

o    Rarely

o    Never

15.  How satisfied are you with your current preparation strategy for OSCE?

o    Very satisfied

o    Satisfied

o    Neutral

o    Unsatisfied

o    Very Unsatisfied

SECTION E: FEEDBACK AND RECOMMENDATIONS

1.      What challenges do you encounter while preparing for OSCE?

o                                                                                                                                                                                          

2.      What resources or support would improve your OSCE preparation?

o                                                                                                                                                                                          

3.      What specific areas of OSCE do you find most challenging?

o                                                                                                                                                                                          

4.      How do you think OSCE preparation can be better integrated into the medical curriculum?

o                                                                                                                                                                                          

5.      What recommendations do you have for the future development of OSCE?

The above questionnaire is made and formatted originally with the help of experts.
